# Discourse on COVID-19 Mass Testing vs. Rapid Testing Processing

**DOI:** 10.3389/fpubh.2022.883490

**Published:** 2022-05-10

**Authors:** Ali Cheshmehzangi, Tong Zou

**Affiliations:** ^1^Network for Education and Research on Peace and Sustainability (NERPS), Hiroshima University, Hiroshima, Japan; ^2^Faculty of Science and Engineering, University of Nottingham Ningbo China, Ningbo, China

**Keywords:** COVID-19, mass testing, rapid processing, rapid testing, containment, test results

## Introduction

COVID-19 “mass testing” and “rapid processing” are often mistaken as one process. While the two are correlated and partly overlapping each other, they are different in terms of processing and implementation. Mass testing is related to the size factor, while rapid processing is mainly related to the time factors. The former is mainly based on testing coverage of a larger population when and where infected clusters are found. At the same time, the latter must be understood as the rapid process of testing and verifying the situation for the suspects of potential positive cases. The two differ in how the pandemic could be contained at smaller or even larger scales. In this opinion article, we delve into this discourse to discuss the differences between the two.

## COVID-19 Mass Testing and What it Entails

As discussed by many scholars (1–4), mass testing is an effective way of identifying infected cases and clusters or hotspots. It helps initiate larger-scale processes such as contact tracing, isolation, and breaking chains of transmission. For example, the combination of mass testing and lockdown can significantly reduce the infected cases by 60% and COVID-19 mortality by 0.41% compared to employing a lockdown strategy alone (5, 6). The belief is that mass testing facilities could end the epidemic rapidly (7). It has been effective for rapid detection and isolation procedures in mini outbreaks of the ongoing COVID-19 pandemic across China (8), UK (9), India (10), and Italy (11).

The governmental function and institutional efficiency in responding to pandemic outbreaks are effective. However, large-scale pandemic control and prevention strategies vary differently across the world. They could depend on the countries' unique contextual factors. Those institutional and political constraints with deteriorating insufficient administrative capacity and weak executive ability can impair the effectiveness of restrictions significantly. Hence, they can cause additional disruptions to other areas like social stability. For instance, a systematic review and meta-analysis (12) found no significant difference in the effect of lockdowns on COVID-19 mortality, with the mortality reduction in Europe and the United States being only about 0.2% for lockdowns and 2.9% for shelter-in-place despite their huge economic and social drawbacks.

While mass testing is not necessarily used to avoid the spread of COVID-19 between different countries and cities, the method was proven to effectively enhance control of the disease spread using intensive contact tracing in a South Korean case study (13). Mass testing processes have enabled countries and cities to work with existing public health infrastructures or propose new facilities to support larger-scale testing. Thus, it is beneficial to public health (14) and effective in tracking close contacts, identifying infected cases in clusters, and finding ways of closing or restricting infected areas. Despite the claims that mass testing is related to rapid finding (15), we argue this is not entirely true.

The main differences between mass testing and the rapid testing process are their focuses. Mass testing is named after the extensiveness and wide coverage of the testing process, while rapid testing process is named for its merits on the speed and efficiency of the entire process. Some rapid testing processes are voluntary, not only useful for people who need to provide a negative report to enter certain public places or transportations, but also essential for patients who need an urgent hospitalization to clear any suspicion of COVID-19 infection. On the other hand, for most circumstances, mass testing processes are mandatory and usually designated by local governments for contamination detection and control. Mass testing is related to scale and is affected by time. Rapid processes and technologies are essential to ensure the effectiveness of mass testing, which are still widely missing. As highlighted by Peto et al. (16), there are indeed unnecessary obstacles to COVID-19 mass testing, which must be looked at from a combination of scientific, governance, and management perspectives. More importantly, mass testing processes are generally costly and time-consuming. Some countries have implemented other methods of pooled testing (17), data collection fin a mass-testing setting (18), mass screening (6), and leading to digital contact tracing (19) and other processes. In earlier days, such an approach was effective after a single suspected or confirmed case was found (20) or in places where border closures and high-level restrictions are still in place.

Because the newer COVID-19 variants Delta and Omicron have faster infection rates, it has become even more important for mass testing to employ rapid processes to be effective. To date, comparative studies of mass testing with tracing and other processes in the UK have identified low-level – yet promising – evidence concerning the effectiveness of mass testing and contract tracing processes (21). Nonetheless, the key arguments are that mass testing alone is ineffective and becomes more effective when combined with other processes or practices, such as contact tracing and lockdown (5, 6). Sold as a rapid testing process, examples such as the UK's Operation Moonshot mass testing programme (22) was not necessarily compelling despite the efforts of weekly testing and creating the so-called “vital loop” to control the disease spread. In this programme, despite the high expenditures, the performance of tests was not effective enough. Poor detection rates and lack of other measures remain questionable factors that show the limitations of such mass testing processes. The process has been different in places where large-scale lockdown and closures are immediately implemented when hotspots are found. For instance, in December 2021, a northern district of the City of Ningbo, East China, was entirely amputated from regular city operations and connections to other parts (of the city). Back then, the city managed to only succeed with mass testing practice just because of the immediate closure of the whole Zhenhai district, after the first case was found on the 6th of December 2021. After reaching the peak in about 2 weeks and the gradual process of smaller-scale containment, the lockdown restrictions were eventually lifted, and mass testing was gradually stopped. To put this in another perspective, we could imagine the ineffectiveness of mass testing if the district was not immediately closed.

Accordingly, existing evidence is not sufficient to prove the effectiveness of mass testing alone in preventing the spreading of COVID-19 disease. But perceptions like “mass testing cannot prevent the disease from spreading” should be avoided. In fact, mass testing could lead to the development of illogical processes and/or redundant routines just to follow governmental regulations and policies, eventually turning into a pointless act of formalism of normalizing pandemic control and prevention. We have seen from many global examples that regular weekly or biweekly mass testing processes have not avoided disease spread but are just used to detect infected cases. Therefore, we argue that mass testing alone cannot be used for containment and ending the pandemic.

There are many limitations, barriers, and constraints regarding mass testing and public health strategy implementation, varying from social, cultural, institutional, technical factors. As a public health strategy, conducting mass testing may encounter social and cultural obstacles like lack of knowledge about the virus, poor understanding of the need for pandemic prevention/poor safety culture, the culture of denial, and/or public stigmatization (23, 24). Other systematic and institutional constraints may involve an underdeveloped healthcare system (23), poor communication between governmental health institutions and the public, lack of administrative commitment and support at the community level, lack of strict enforcement of regulations, and lack of resources and funds (24). Furthermore, one crucial factor that needs careful attention in mass testing practices is the quantity and quality of testing kits and technologies. In most places, facilities cannot necessarily handle mass testing. With higher demand, we often see difficulties following the safety and control measures and protocols meant to keep people safe. In mass testing procedures, large-scale groups of people are lined up in clusters where the risk of getting infected is even higher. Some innovative methods, such as the use of biosensors (25), are proposed to change the landscape of mass testing procedures, but are yet not implemented or are still experimental. Some have questioned the accuracy of rapid testing; however, mass spectrometry-based detection of COVID-19 host response has a reported sensitivity and specificity of 100 and 93% respectively in a diverse population including those who are asymptomatic, have been vaccinated for COVID-19, and who have any COVID-19 variant (26). This emphasizes another missing factor in mass testing processes: time.

The temporal aspect plays a significant part in the effectiveness of mass testing, making the argument of mass testing vs. rapid testing processing valid for future research directions. What we see globally are mass testing procedures or mass testing combined with restricted measures. The latter has been more effective but lacks rapid identification and containment. Thus, we urge to consider what has been discussed beyond just the scaling up testing capacity (27) and toward genuine rapid testing processes and/or practices. In this regard, the use of more advanced technologies cannot be disregarded, meaning that we have not yet explored other effective alternatives (28–30). Thus, mass testing could only cover testing a larger population. Without closure restrictions, the approach is merely costly to governments and fatiguing for our already overexploited public health facilities and services. An example is that if a person involved in mass testing or regular testing could travel from A to B without a problem, then there are obvious flaws in this process. If the test results are not out before the person's departure to another location, then the testing was not done to avoid the spread of the disease but to detect if the person is infected or is contacted with an infected person. Hence, mini outbreaks keep reoccurring just because the test results are delayed for several hours and sometimes up to a whole 24 h cycle. This lack of rapid processing leads to the development of absurd formal processes of regular testing without understanding the importance of rapid test results. This fact puts a critical question on mass testing effectiveness if rapid processing is not considered or embedded in such practices.

## Discussion: Mass Testing vs. Rapid Processing of Testing

Since mobility causes the rapid spread of the disease (31, 32), we cannot just rely on current mass testing methods to end the pandemic. Without suitable frequency, speedy efficiency, and effective protocols to ensure and confirm all the positive suspects within the least time frame regarding the transmissibility timeline, most efforts of mass testing are very likely to be wasted. For instance, undetected active infections can be developed into mini outbreaks (33) or even larger very quickly through a contaminated airplane (34, 35). Ongoing research studies on rapid processing of testing highlight the urgent need for novel testing techniques beyond just scale and more related to the faster processing of tests (27). While testing strategies differ from country to country, we have yet to see which testing model has been more effective in the long run. The wide-scale regular community testing processes could only be effective if rapid processing is embedded in their processes. Otherwise, breaking chains of transmissions becomes a mission impossible, and this pandemic could be further prolonged. An example is to have rapid and accurate testing processing on departure or arrival points, to ensure test results are out before people's departure or entry to different locations (e.g., cities, countries, etc.). It is already evidenced that even a 72 h test result with several weeks of restricted isolation and quarantine does not solve the problem of disease spread (36, 37). Yet, rapid testing remains challenging (38), or else they could curb COVID-19 much earlier. Despite their current challenges, rapid antigen tests have shown to be promising in smaller scales (39), meaning that regular processes are not the only way of keeping communities safe.

Lastly, we note that COVID-19 mass testing protocols are still weak. They only provide support to rapid response in the cases of detection and isolation but are not helping to avoid the spread of the disease. For example, to deal with the potential risks of false negatives, protocols for repeated testing and isolations of patients with a single negative result but COVID-19 symptoms are suggested (40). Therefore, frequent mass testing without rapid processing of the results is a costly process by all means. The current and future research should focus on the deployment and utilization of faster and more accurate technologies to ensure test results are processed rapidly. It is only then that mass testing could save us from the ongoing pandemic, as we would be able to detect, isolate, and treat the infected person in his/her original location of A, and not when he/she has already arrived at point B. This pandemic has proven that “time” is a crucial factor should we wish to reach an end to this prolonging adversity any time soon.

## Author Contributions

AC drafted and wrote the paper. TZ worked on literature review and revisions. Both authors contributed to the article and approved the submitted version.

## Conflict of Interest

The authors declare that the research was conducted in the absence of any commercial or financial relationships that could be construed as a potential conflict of interest.

## Publisher's Note

All claims expressed in this article are solely those of the authors and do not necessarily represent those of their affiliated organizations, or those of the publisher, the editors and the reviewers. Any product that may be evaluated in this article, or claim that may be made by its manufacturer, is not guaranteed or endorsed by the publisher.
